# Genetic association of ACE2 and TMPRSS2 polymorphisms with COVID-19 severity; a single centre study from Egypt

**DOI:** 10.1186/s12985-024-02298-x

**Published:** 2024-01-23

**Authors:** Marwa H. Elnagdy, Alshimaa Magdy, Waleed Eldars, Mohamed Elgamal, Ahmed Hazem El-Nagdy, Omnia Salem, Mohamed Magdy Elmowafy, Omar Ahmed Elborsh, Abdelrahman Walid Elshafey, Muhammad Magdy Kesba, Ahmed Elsaeed Abdulgalil, Ali Sobh

**Affiliations:** 1 Department of Medical Biochemistry and Molecular Biology, Mansoura University Faculty of Medicine, Mansoura, Egypt; 2 Department of Basic Medical Sciences, Faculty of Medicine, New Mansoura University, Mansoura, Egypt; 3 Department of Medical Microbiology and Immunology, Mansoura University Faculty of Medicine, Mansoura, Egypt; 4 Department of Chest Medicine, Mansoura University Faculty of Medicine, Mansoura, Egypt; 5 Department of Microbiology, Faculty of Dentistry, Horus University, Damietta El Gadeeda, Egypt; 6 Department of Pediatrics, Mansoura University Children’s Hospital, Mansoura University Faculty of Medicine, 60 El Gomhouria Street, Mansoura, 35516 Egypt; 7 Intern, Mansoura University Hospitals, Mansoura University, Mansoura, Egypt; 8 Neurology Resident at Fayoum General Hospital, Faiyum, Egypt; 9 Mansoura Nephrology and Dialysis Unit, Internal Medicine Department, Mansoura University Faculty of Medicine, Mansoura, Egypt

**Keywords:** ACE2, TMPRSS2, Polymorphism, COVID-19, Genotyping, Egypt

## Abstract

**Background:**

Since the emergence of the COVID-19 infection in China, it has caused considerable morbidity, mortality, and economic burden. It causes the vast majority of clinical manifestations, ranging from mild or even no symptoms to severe respiratory failure. There are many risk factors for severe COVID-19, such as old age, male gender, and associated comorbidities. A major role for genetic factors may exist. The SARS-CoV-2 virus enters the cell primarily through ACE2 receptors. rs2285666 is one of many polymorphisms found in the *ACE2* receptor gene. To enable endosome-independent entry into target cells, the transmembrane protease serine-type 2 (TMPRSS2) is necessary to cleave the virus’ spike (S) glycoprotein. *TMPRSS2* is characterized by an androgen receptor element. The rs12329760 polymorphism in *TMPRSS2* may explain different genetic susceptibilities to COVID-19.

**Method:**

This cross-sectional study was held in Mansoura University Hospitals during the period from June 2020 to April 2022 on patients who had mild and severe COVID-19. Demographic, clinical, and laboratory data were collected, and the TaqMan real-time polymerase chain was used for allelic discrimination in the genotyping of rs2285666 and rs12329760.

**Results:**

This study included 317 Egyptian patients, aged from 0.2 to 87 years. Males were 146, while females were 171. They were divided into mild and severe groups (91 and 226 patients, respectively) based on their clinical symptoms. There was a significant association between COVID-19 severity and male gender, hypertension, diabetes mellitus, and high CRP. The genotype and allele frequency distributions of the *ACE2* rs2285666 polymorphism showed no significant association with the severity of COVID-19 in both. In contrast, in *TMPRSS2* rs12329760 minor T allele and CT, TT genotypes were significantly associated with a reduced likelihood of developing severe COVID-19.

**Conclusion:**

Our study indicates that the *ACE2* rs2285666 polymorphism is not related to the severity of COVID-19, whether genotypes or alleles. In *TMPRSS2* rs12329760, the dominant model and T allele showed significantly lower frequencies in severe cases, with a protective effect against severity. The discrepancies with previous results may be due to variations in other ACE2 receptor-related genes, inflammatory mediators, and coagulation indicators. Haplotype blocks and differences in racial makeup must be taken into consideration. Future research should be done to clarify how ethnicity affects these polymorphisms and how other comorbidities combine to have an additive effect.

## Introduction

One of the deadliest pandemics in the past hundred years was the Coronavirus Disease 2019 (COVID-19) pandemic [1]. By January 2023, there were more than 670 million infections and more than 6.5 million fatalities worldwide [2]. The severe acute respiratory syndrome coronavirus-2 (SARS-CoV-2), responsible for COVID-19, has spread quickly and steadily, affecting human health and the stability of the global economy [3]. This has triggered a crisis that has spread across the globe. The clinical spectrum of SARS-CoV-2 infection is wide, ranging from asymptomatic or mildly symptomatic to severe symptoms that require admission to intensive care [4].

The known risk factors for increased COVID-19 morbidity and mortality include older age, male gender, and associated comorbidities like diabetes, obesity, and cardiovascular disease. Another important risk factor for COVID-19 is the influence of a person’s genetics [4]. The discovery of host genetic pathways and DNA polymorphisms that regulate the risk of infection and disease severity will significantly aid in the development of new COVID-19 preventive and/or therapeutic strategies [5].

SARS-CoV-2 utilizes the angiotensin-converting enzyme 2 (ACE2) receptor for entry into the cells and the host transmembrane serine protease (TMPRSS2) for S protein priming [6, 7]. Therefore, the study of polymorphisms in *ACE2* and *TMPRSS2* in various populations could open the way for precision medicine and individualized COVID-19 treatment plans [8].

ACE2 is a type I transmembrane enzyme with homology to ACE, which plays a key role in the Renin-Angiotensin system and is a target for the treatment of hypertension [9]. ACE2 receptors are the main host cell receptors responsible for viral entry into the cell [6]. This occurs by binding of viral spike glycoprotein to ACE2 receptors of the host cells [10].

It was hypothesized that higher susceptibility to COVID-19 infection is related to the expression of the target ACE2 receptor in the epithelium exposed to the virus [11]. Age affects the expression of the *ACE2* receptor gene in the nasal epithelium. which is the first site of SARS-CoV2 contact [12]. The lower expression of the ACE2 receptor in children may explain the reduced risk [13]. However, ACE2 expression in the oral cavity mucosa may enable the virus to cause infection more easily [14]. Smoking and chronic obstructive pulmonary disease have been shown to increase the expression of ACE2 receptors in the lower respiratory tract and thus the risk of COVID-19 infection [15, 16].

The expression level of *ACE2* receptor gene is largely influenced by genetic variations. rs2285666 is one of the numerous polymorphisms found in the *ACE2* receptor gene [17]. It is located in the third intron’s fourth base and the intron next to the exon, and it can change messenger RNA alternate splicing and affect the expression of the *ACE2* receptor gene [18]. It had population-based frequency differences [19].

The transmembrane protease serine-type 2 (TMPRSS2) plays a significant role in coronavirus infections. It is necessary for priming the glycoprotein of the virus spike by its cleavage for easier entry into target cells in an endosome-independent way [6]. There are androgen receptor elements upstream of the transcription site of *TMPRSS2* [20]. Type I alveolar epithelial cells and ciliated cells were found to have the highest levels of TMPRSS2 expression [21]. Additionally, it is co-expressed with ACE2 [22], which is the SARS-CoV-2 cellular receptor [15].

SARS-CoV-2 infection has been shown to be inhibited in vivo by *TMPRSS2* knockout [23]. This was associated with a diminished pro-inflammatory viral response [24]. Studies conducted in vitro have demonstrated that TMPRSS2 inhibitors protect against SARS-CoV-2 infections of primary airway cells. Mice infected with SARS-CoV and given the serine protease inhibitor survived [25]. Based on these results, it was proposed that a genetic change in *TMPRSS2* may have an impact on the severity of the infection. The rs12329760 polymorphism in *TMPRSS2* may play an important role [8].

Considering the role of ACE2 and TMPRSS2 in COVID-19 pathogenesis and the variation in disease severity, rs2285666 and rs12329760 polymorphisms have attracted attention. Since there were discrepancies between previous results, which may be attributed to host factors, including ethnicity, we aimed to study the polymorphisms of rs2285666 and rs12329760 in COVID-19-positive Egyptian patients and their relationship to the severity of the disease. This may pave the way for precision medicine and personalized treatment strategies for COVID-19.

## Patients and methods

### Study population

This cross-sectional study included 317 Egyptian patients with SARS-CoV-2 infection confirmed by RT-PCR testing in at least one biological sample. Mild cases included 91 patients, while severe cases included 226 patients. Severity of COVID-19 cases was considered with a diagnosis of viral pneumonia or myocardial infarction within 14 days after the SARS-CoV-2 positive test, hospitalization for 7 days or longer, or intensive care unit admission with clinical and laboratory findings suggesting a decrease in oxygen saturation, respiratory distress, and signs of pneumonia according to World Health Organization (WHO) severity guidelines [26]. Mild cases exhibited signs and symptoms like loss of taste and odor, dry cough, exhaustion, fever, diarrhea, chills, nasal congestion, sore throat, conjunctivitis, headache, musculoskeletal pain, skin rashes, and dizziness with a history of COVID-19 contact and confirmed by positive PCR. All severe cases who were admitted to the isolation unit at Mansoura University Hospitals during the study period were included, while mild cases were obtained from medical residents, laboratory personnel, nurses, and employees who had close contact with COVID-19 cases. This study was conducted at the Department of Medical Biochemistry and Molecular Biology, Faculty of Medicine, Mansoura. Informed consent was obtained from all study participants or their relatives. The Institutional Research Board approved the protocol (RP.20.05.70). A complete, comprehensive medical history was obtained, and a complete clinical examination was performed, including age, disease duration, comorbidities, signs of infection, and complications.

### Laboratory analyses

The blood samples were collected during the period from June 2020 to April 2022. Samples were processed cautiously, and laboratory parameters were assessed, including CBC, C-reactive protein (CRP), ferritin, D-dimer, AST (aspartate aminotransferase), ALT (alanine aminotransferase), and creatinine.

### Genetic analysis

**Fig. 1:**
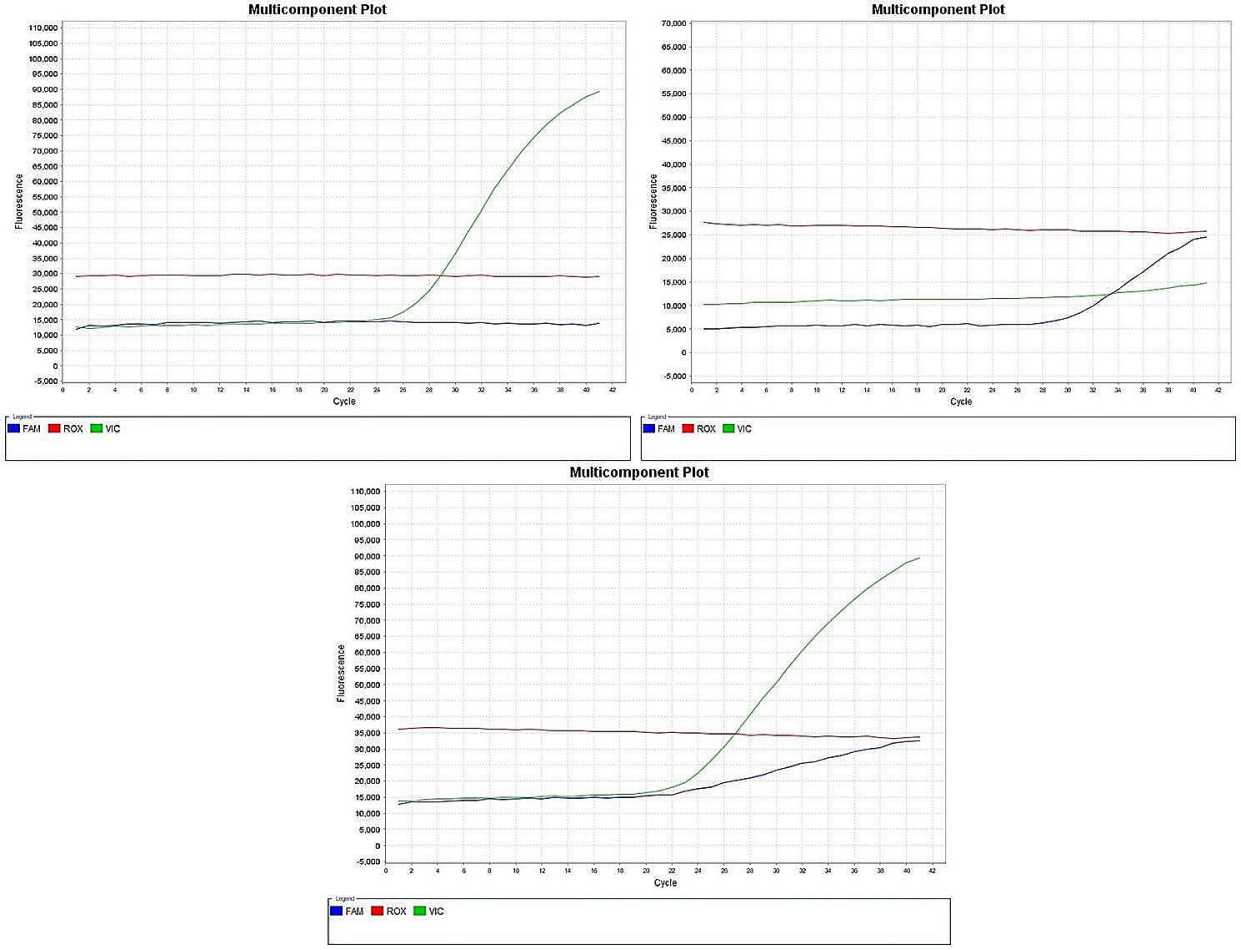
Real-time polymerase chain reaction of the three genotypes of ACE2 (rs2285666): (a) homozygous CC wild-type genotype, (b) homozygous TT variant genotype, (c) heterozygous CT genotype. The multicomponent plot analysis displays the result of each sample based on the dye released. For example, when the VIC green dye is released, this indicates a homozygous wild-type genotype. The release of FAM blue dye alone refers to homozygous variant genotype. In the case of the release of both dyes, this refers to heterozygosity for both alleles

**Fig. 2:**
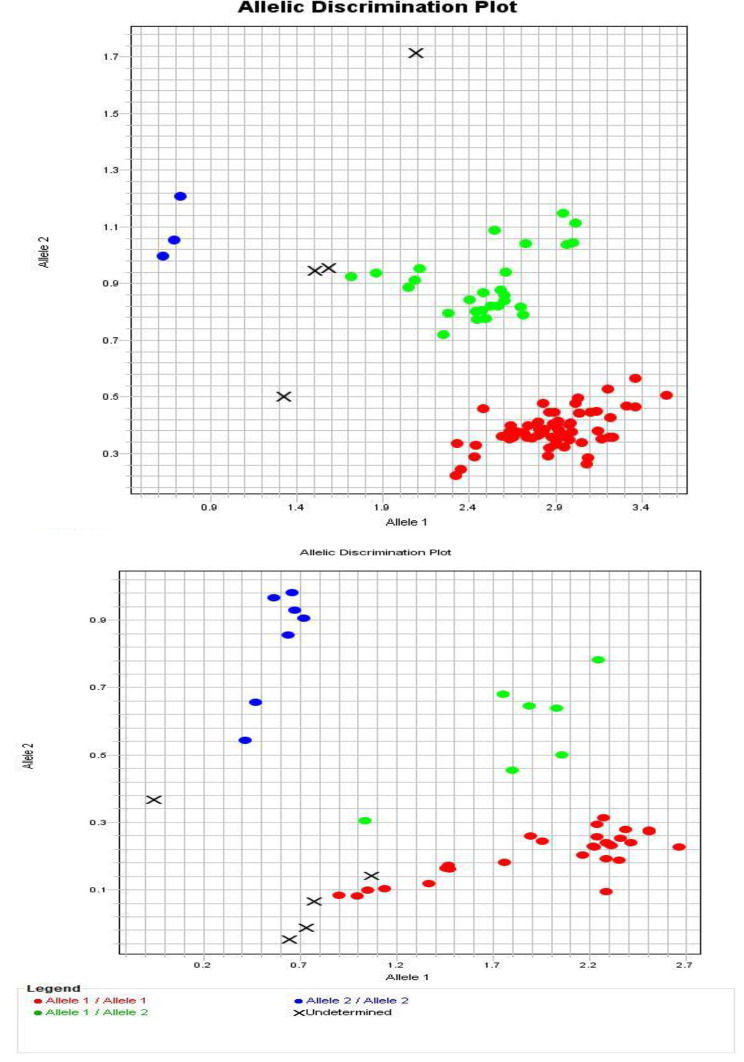
Allelic discrimination plot of the three genotypes of *ACE2* C/T (rs2285666) and *TMPRSS2* C/T (rs12329760) respectively. Red dots represent homozygous CC wild-type genotype, blue dots for homozygous TT variant genotype, green dots for heterozygous CT genotype

Two milliliters of each subject’s blood were drawn via venipuncture and put into an ethylene diamine tetraacetic acid (EDTA) tube. Until DNA extraction, blood was kept at -20°. Genomic DNA was extracted from blood samples using the QIAamp DNA Extraction Micro Kit (Qiagen, Germany) according to the manufacturer’s instructions. The NanoDrop 2000c Spectrophotometer from Thermo Scientific (USA) was used to assess DNA concentration and purity. The DNA purity of the examined samples is accepted if it ranges from 1.6 to 1.9. This study focused on genotyping of two SNPs, *ACE2* C/T (rs2285666) and *TMPRSS2* C/T (rs12329760). This was performed using pre-designed TaqMan SNP genotyping assays (Thermo Fisher Scientific, C___2551626_1_ and C__25622353_20, respectively). Each assay contains two distinct forward and reverse primers that flank each SNP, and two TaqMan probes each of which was labeled with a fluorescent dye (either VIC or FAM) that differed only at the SNP site (Table 1). One probe was complementary to the wild-type allele and the other to the variant allele (Figs. 1 and 2). This was done by allelic discrimination using TaqMan real-time polymerase chain instrument (Azure Cielo 6, Azure, USA).

**Table 1:** TaqMan genotyping assay:

SNP ID	Gene	Gene Name	Context Sequence [VIC/FAM]	VIC	FAM
rs2285666	ACE2	Angiotensin I converting enzyme 2	ATAATCACTACTAAAAATTAGTAGC**[C/T]**TACCTGGTTCAAGTAATAAGCATTC	C	T
rs12329760	TMPRSS2	Transmembrane protease, serine 2	CAGGACTTCCTCTGAGATGAGTACA**[C/T]**CTGAAGGATGAAGTTTGGTCCGTAG	C	T

### Statistical analysis

Statistical analysis of the data was done using Statistical Package for Social Science (SPSS) version 21 and SNP Stats software. To compare qualitative data, the Chi-square test was employed. The Mann-Whitney U test was used as a test of significance for the comparison of the two groups. The quantitative data were expressed as the median and interquartile range (IQR). Odds ratios (OR), *P* values, and 95% confidence intervals (CI) were used to present the data. Results were regarded as statistically significant if the *p*-value was < 0.05.

## Results

Out of 317 COVID-19 patients enrolled in this study; 146 were males and 171 were females. Age ranged from 0.2 to 87 years (Table 2). They were classified into mild and severe patients (91 and 226, respectively). Table 3 demonstrates that males experienced a greater increase in disease severity than females (*P* < 0.001). Severity also increased with history of COVID contact, hypertension, and DM and it was significantly associated with hospitalization, oxygen therapy, ICU admission, mechanical ventilation, respiratory distress, pneumonia, ARDS, shock, sepsis, multi-organ failure, acute kidney injury, and hepatitis and seizures (*p* < 0.05) (Table 4). Severe cases were also significantly associated with anemia, leukocytosis, neutrophilia, lymphopenia, decreased platelet count, increased CRP, D-dimer, ALT, and creatinine (*p* < 0.001) (Table 5). The association between the two examined SNPs and the severity of COVID-19 was displayed in Table 6. Regarding rs2285666, males and females were analyzed in a separate way because of the location of *ACE2* receptor gene on X chromosome and males are hemizygous for *ACE2*. Neither genotypes nor allele frequencies were statistically associated with COVID-19 severity in rs2285666. Regarding rs12329760, the dominant model and T allele showed significantly lower frequency in severe cases, with a protective effect against severity.

**Table 2:** Age and gender among studied cases

		Total *N* = 317	
Age (median, min-max)		43	0.2–87
Sex (*N*, %)	**Male**	146	46.1
	**Female**	171	53.9

**Table 3:** Association of gender with severity

	Sex				
	Male *N* = 146		Female *N* = 171		*P*
	*N*	%	*N*	%	
**Mild**	26	17.8%	65	38.0%	**< 0.001**
**Severe**	120	82.2%	106	62.0%	

**Table 4:** Clinical data among studied cases

	Total *N* = 317		Mild *N* = 91		Severe *N* = 226		*P*
	*N*	%	*N*	%	*N*	%	
Covid Contact	185	58.9%	20	22.0%	165	74.0%	**< 0.001**
HTN	126	40.1%	16	17.6%	110	49.3%	**< 0.001**
DM	86	28.0%	5	5.5%	81	37.5%	**< 0.001**
Chronic Lung Disease	9	2.9%	3	3.3%	6	2.7%	0.721
Asthma	7	2.2%	2	2.2%	5	2.2%	1
CKD	5	1.6%	0	0.0%	5	2.2%	0.326
CLD	3	1.0%	0	0.0%	3	1.3%	0.560
Recurrent infections	1	0.3%	0	0.0%	1	0.4%	1
Malignancy	8	2.5%	3	3.3%	5	2.2%	0.694
Autoimmune/Collagen disease	3	1.0%	0	0.0%	3	1.3%	0.560
Immusuppressive drugs	3	1.0%	0	0.0%	3	1.3%	0.560
Hospitalization	223	71.2%	1	1.1%	222	100.0%	**< 0.001**
Oxygen therapy	179	57.2%	1	1.1%	178	80.2%	**< 0.001**
ICU admission	125	39.9%	0	0.0%	125	56.3%	**< 0.001**
Mechanical Ventilation	83	26.8%	0	0.0%	83	37.9%	**< 0.001**
Respiratory distress	182	58.1%	37	40.7%	145	65.3%	**< 0.001**
Pneumonia	207	65.7%	0	0.0%	207	92.4%	**< 0.001**
ARDS	158	50.2%	0	0.0%	158	70.5%	**< 0.001**
Shock	6	3.7%	0	0.0%	6	8.3%	**0.007**
Sepsis	97	31.0%	0	0.0%	97	43.7%	**< 0.001**
MOF	47	15.0%	0	0.0%	47	21.1%	**< 0.001**
AKI	40	12.7%	0	0.0%	40	17.9%	**< 0.001**
Hepatitis	64	20.4%	1	1.1%	63	28.3%	**< 0.001**
Stroke	10	3.2%	0	0.0%	10	4.5%	0.069
Seizures	12	3.8%	0	0.0%	12	5.4%	**0.022**

Abbreviations: DM: Diabetes Mellitus, CKD: Chronic Kidney Disease, CLD: Chronic Liver Disease, ICU: Intensive Care Unit, ARDS: Acute Respiratory Distress Syndrome, MOF: Multi-Organ Failure, AKI: Acute Kidney Injury

**Table 5:** Laboratory parameters among studied cases

	Total *N* = 317			Mild *N* = 91			Severe *N* = 226			*P*
	Median	min-max		Median	min-max		Median	min-max		
HB g/dL	11.4	3.0	17.7	12.2	8.7	16.2	10.6	3.0	17.7	**< 0.001**
Total leukocyte Count k/Ul	11.3	1.4	44.6	9.5	3.0	18.0	13.9	1.4	44.6	**< 0.001**
Neutrophils k/uL	7.9	0.4	40.3	6.3	1.1	12.0	10.3	0.4	40.3	**< 0.001**
Lymphocyte k/uL	1.2	0.1	9.2	2.5	0.4	6.7	0.9	0.1	9.2	**< 0.001**
Platelets k/uL	203.0	13.0	832.0	241.0	169.0	832.0	168.0	13.0	737.0	**< 0.001**
C.R.P	58.0	1.0	434.0	3.0	1.0	5.0	109.0	1.0	434.0	**< 0.001**
Ferritin	532.0	15	2000.0	-	-	-	532.0	15	2000.0	0.111
D-Dimer	10.2	0.1	13.2	0.2	0.2	0.2	10.2	0.1	13.2	**< 0.001**
AST	36.0	12.0	6407.0	32.0	20.0	91.0	40.0	12.0	6407.0	0.526
ALT	42.0	4.0	4202.0	43.0	6.0	136.0	39.0	4.0	4202.0	**< 0.001**
Creatinine	0.7	0.3	20.7	0.6	0.3	1.3	0.9	0.3	20.7	**< 0.001**

Abbreviations: HB: Hemoglobin, CRP: C-Reactive Protein, ALT: Alanine aminotransferase. AST: aspartate aminotransferase

**Table 6:** Association of studied SNPs with COVID-19 severity

			Mild *N* = 91		Severe *N* = 226		*p*	OR	95% CI
			*N*	%	*N*	%			
**rs2285666** **females**	**Genotypes**	**CC**	42	64.6%	57	53.8%	-	1	Reference
		**CT**	13	20.0%	25	23.6%	0.379	1.241	0.768–2.005
		**TT**	10	15.4%	24	22.6%	0.178	1.420	0.853–2.362
	**Dominant**	**CC**	42	64.6%	57	53.8%	-	1	Reference
		**CT + TT**	23	35.4%	49	46.2%	0.163	1.321	0.894–1.952
	**Recessive**	**CC + CT**	55	84.6%	82	77.4%	-	1	Reference
		**TT**	10	15.4%	24	22.6%	0.246	1.339	0.818–2.191
	**Alleles**	**C**	97	74.6%	139	65.6%	-	1	Reference
		**T**	33	25.4%	73	34.4%	0.078	1.306	0.971–1.758
**rs2285666** **males**	**Alleles**	**C**	19	20.9%	89	39.4%	-	1	Reference
		**T**	7	7.7%	31	13.7%	0.909	1.032	0.602–1.771
**rs12329760**	**Genotypes**	**CC**	59	64.8%	172	76.1%	-	1	Reference
		**CT**	19	20.9%	30	13.3%	0.066	0.689	0.463–1.025
		**TT**	13	14.3%	24	10.6%	0.230	0.759	0.484–1.190
	**Dominant**	**CC**	59	64.8%	172	76.1%	-	1	Reference
		**CT + TT**	32	35.2%	54	23.9%	**0.044**	0.718	0.520–0.991
	**Recessive**	**CC + CT**	78	85.7%	202	89.4%	-	1	Reference
		**TT**	13	14.3%	24	10.6%	0.364	0.814	0.523–1.268
	**Alleles**	**C**	137	75.3%	374	82.7%	-	1	Reference
		**T**	45	24.7%	78	17.3%	**0.034**	0.759	0.588–0.979

OR, odds ratio; CI, confidence interval. T is the minor allele in both SNPs.

The current study showed an interesting finding which is the gender-specific differential effect of rs12329760 alleles. A sexual dimorphic effect in the genetic association of rs12329760 with the severity of COVID-19 was noticed. In males, the rs12329760 T allele was significantly associated with more severe cases (*p* = 0.014, 95% CI: 1.124–2.801), while in females, the rs12329760 C allele was associated with more severe cases and the presence of T allele seems to be protective (*p* < 0.001, 95% CI: 0.198–0.448). The rs2285666 was not significantly associated with severity among males and females (Table 7).

Regression analysis was conducted for the prediction of COVID-19 severity. Older age, male gender, presence of comorbidities, and high CRP, were associated with the risk of severe cases, while the rs12329760 dominant model was associated with a protective effect against COVID-19 severity in univariable analysis. However, in multivariable analysis, only older age, male gender, presence of comorbidities, and a high CRP were considered as risk predictors of COVID-19 severe cases (Table 8).

**Table 7:** Association of studied SNPs with gender and severity

			Mild		Severe		*p*	OR	95% CI
			*N*	%	*N*	%			
**Males**	**rs2285666**	**C**	19	73.1%	89	74.2%	-	1	Reference
		**T**	7	26.9%	31	25.8%	0.909	0.969	0.565–1.662
	**rs12329760**	**CC**	20	76.92%	74	61.67%	-	1	Reference
		**CT**	6	23.08%	26	21.67%	0.759	1.094	0.614–1.950
		**TT**	0	0%	20	16.67%	1	-	-
		**C**	46	88.5%	174	72.5%	-	1	Reference
		**T**	6	11.5%	66	27.5%	**0.014**	1.774	1.124–2.801
**Females**	**rs2285666**	**CC**	42	64.62%	57	53.77%	-	1	Reference
		**CT**	13	20.00%	25	23.58%	0.379	1.241	0.768–2.005
		**TT**	10	15.38%	24	22.64%	0.178	1.420	0.853–2.362
		**C**	97	74.6%	139	65.6%	-	1	Reference
		**T**	33	25.4%	73	34.4%	0.078	1.306	0.971–1.758
	**rs12329760**	**CC**	39	60.00%	98	92.45%	-	1	Reference
		**CT**	13	20.00%	4	3.77%	**< 0.001**	0.275	0.138–0.550
		**TT**	13	20.00%	4	3.77%	**< 0.001**	0.275	0.138–0.550
		**C**	91	70.0%	200	94.3%	-	1	Reference
		**T**	39	30.0%	12	5.7%	**< 0.001**	0.298	0.198–0.448

**Table 8:** Regression analysis for prediction of COVID severity

	Univariable				Multivariable						
	*P*	OR	95% CI		*P*		OR		95% CI		
Age	**0.021**	1.008	1.001	1.015	**< 0.001**		1.954		1.933		1.976
Female versus male	**0.005**	0.599	0.421	0.854	**0.024**		0.441		0.217		0.896
Comorbidities	**< 0.001**	2.351	1.628	3.396	**0.007**		3.555		1.423		8.880
CRP	**< 0.001**	1.295	1.135	1.479	**< 0.001**		1.370		1.173		1.600
rs12329760 dominant model	**0.044**	0.718	0.520	0.991	0.232		1.584		0.745		3.368
rs12329760 (T)	**0.034**	0.759	0.588	0.979		0.84		1.17		0.24	5.65
rs2285666 in males	0.909	1.032	0.602	1.771	-		-		-		-
rs2285666 in females	0.163	1.321	0.894	1.952	-		-		-		-

OR, odds ratio; CI, confidence interval

## Discussion

COVID-19 is the second pandemic in the twenty-first century, accounting for more than 100 million cases and more than two million fatalities [27]. On August 20th, 2022, the number of documented patients in Egypt was 515,198, with nearly 24,786 deaths [28]. Variation in the severity of COVID-19 can be partially explained by the genetic background of the host and other risk factors like age, gender, and underlying clinical conditions [2]. The analysis of about 81,000 human genomes suggests a possible association between susceptibility, severity, and clinical outcomes of COVID-19 and *ACE2* and *TMPRSS2* DNA polymorphisms. This may help to explain related epidemiological observations and direct the individualized treatment of COVID-19 [8].

It was reported that infection with SARS-CoV-2 is related to the male gender [29–31]. Our results advocate the association between the severity of COVID-19 and the male gender. Similarly, Jin et al. (2020) found that males with COVID-19 are more likely to experience poorer outcomes, regardless of age [32]. However, Alimoradi et al. (2022) reported that the incidence and severity of COVID-19 infection were not significantly related to gender [33]. Higher incidences of hypertension and diabetes mellitus were significantly correlated with COVID-19 severity, and this is in line with studies from China and Italy that proved they are the most prevalent comorbidities associated with SARS-CoV-2. These co-morbidities were known to be linked to ACE2 deficiency [33, 34], which is possibly an effect of glycosylation in diabetes mellitus [35] and maybe a causative factor for hypertension [36].

The severity of COVID-19 may be affected by a high nasopharyngeal viral load or by the host’s immune response. A high viral load and diminished virus-shedding are related to severe COVID-19. This leads to macrophage activation syndrome and cytokine storm [37, 38]. This is in accordance with our results, which showed a significant increase in inflammatory markers (CRP, ferritin, and D-dimer) in severe cases versus mild. Pro-inflammatory cytokine overproduction worsens acute respiratory distress syndrome and causes extensive tissue damage that eventually causes death by causing multiple organ failure [17]. We also found a significant decrease in lymphocyte number in severe cases. This confirms the results of Chen et al. (2020) and may be explained by the ability of SARS-CoV-2 to inhibit hematopoiesis in the bone marrow [39].

rs2285666 is a possible risk factor for type 2 diabetes, hypertension, and coronary artery disease [19]. The significant relationship between these variables and COVID-19 severity in our study supports the idea that rs2285666 may be a predisposing factor associated with the comorbidities seen in COVID-19 patients. The prevalence and risk of SARS-CoV-2 infection in the Indian, Caucasian, and Iranian populations were significantly correlated with the wild genotype of variant rs2285666 [3, 33]. A lower infection rate and case fatality were strongly correlated with the mutant allele in Indian populations [3].

Sequencing of the *ACE2* receptor gene revealed that there is no strong evidence linking variations in the *ACE2* coding sequence to the severity of COVID-19 [40, 41]. However, severity may be affected by the genetic variations in the noncoding regions of the *ACE2* receptor gene or in other noncoding DNAs that control the expression levels of *ACE* genes [42]. The intronic location of the rs2285666 SNP may change mRNA splicing, gene expression, and ACE2 protein levels [36]. However, in our study, we found no significant association between genetic variants of rs2285666 and COVID-19 severity in all groups. This confirms the results of Karakaş Çelik et al. (2021) and Alimoradi et al. (2022), who found no association between rs2285666 and intron variants of the *ACE2* receptor gene [17, 33]. On the contrary, Möhlendick et al. (2021) found that the GG genotype or G-allele was significantly associated with increased severity of SARS-CoV-2 [43]. Moreover, the meta-analysis done by Keikha and Karbalaei (2022) concluded that in people possessing the rs2285666 GG genotype, the risk of progression to severe infection is high, while the rs2285666 GA genotype has a protective role in patients against severe COVID-19 [44].

The susceptibility might not be an *ACE2* receptor gene polymorphism. It may also be influenced by other variables, such as different ethnicities, genders, comorbidities, humidity, density of population, temperature, social isolation, or other polymorphisms. Variations in epigenetic mechanisms related to the expression of *ACE2* receptors may play a role. There may also be a role for histone methylation [23]. Lambert et al. (2008) found that miR421 suppresses the gene of the *ACE2* receptor [42]. The *ACE2* receptor gene is post-translationally modified by phosphorylation and glycosylation [45].

The spread and pathogenesis of coronavirus depend on the activity of the TMPRSS2 enzyme [46]. Black people have an increased burden of COVID-19 [13], and this may be related to the increased nasal expression of TMPRSS2. Bioinformatics methods applied to large public domain datasets identified the rs12329760 in TMPRSS2 as a functionally significant variant in COVID-19 [19, 47]. To explain the high incidence and mortality rate of COVID-19 in the Italian population in comparison to Asian and other European countries, this variant has been put forth as one of the candidate gene variants [19].

*TMPRSS2* gene expression is one of the potential mechanisms explaining the difference in COVID-19 severity in males versus females [48]. Androgen hormones are known to regulate the expression of the *TMPRSS2* gene [20]. Our goal was to ascertain whether there was a connection between the severity of COVID-19 in the Egyptian population and the TMPRSS2 rs12329760 variant.

Our research revealed a significant association between rs12329760, and the severity of COVID-19 and that the T allele showed a significantly lower frequency in severe cases. This is supported by several studies [4, 49–51] that found that the minor T allele of this variant is linked to a decrease in the severity of symptoms of COVID-19, and this is in line with the results of the meta-analysis that showed a significant association between the *TMPRSS2* rs12329760 C-allele and an increased risk of developing severe COVID-19 [46]. However, our results are in contrast to the previous studies in Iran [2, 52] and Egypt [53] that found that the T allele is a risk allele for the severe form of COVID-19. Also, earlier research done in Germany and Indonesia failed to discover any correlation between this *TMPRSS2* gene variant and the severity of COVID-19 [46, 54].

The frequency of each of the studied genotypes and alleles was done in subgroups and showed that the C allele is significantly higher in females with severe infection. In contrast, the T allele was significantly associated with more severe cases in males.

This discrepancy with the results of various studies may be explained by the absence of androgen hormones dependent control of *TMPRSS2* gene expression in lungs [55]. The patient’s ancestry has been postulated as a possible factor in the T allele effect on the severity of COVID-19 [2]. Additionally, variations in ethnicity, the presence of haplotype blocks with a unique combination of other risk loci, and variations of other host factors affecting immunity may have a role.

## Conclusion

Our study concludes there is no evidence linking the *ACE2* rs2285666 to the severity of COVID-19. While the results of the *TMPRSS2* rs12329760 polymorphism showed that the minor T allele and CT and TT genotypes are protective against COVID-19 severity. Additional research is required to explain the gender-specific differential effect of rs12329760 alleles. The differences between our results and other studies may be due to the existence of haplotype blocks and racial differences. The impact of ethnicity on these polymorphisms and their relationship to COVID-19 severity should be clarified in upcoming multiethnic studies.

## Data Availability

The datasets used and/or analyzed during the current study are available from the corresponding author upon reasonable request.
